# The spatial distribution of leprosy cases during 15 years of a leprosy control program in Bangladesh: An observational study

**DOI:** 10.1186/1471-2334-8-126

**Published:** 2008-09-23

**Authors:** EAJ Fischer, D Pahan, SK Chowdhury, JH Richardus

**Affiliations:** 1 Department of Public Health, Erasmus MC, University Medical Center Rotterdam, Rotterdam, The Netherlands; 2 Rural Health Program, Leprosy Mission Bangladesh, Nilphamari, Bangladesh

## Abstract

**Background:**

An uneven spatial distribution of leprosy can be caused by the influence of geography on the distribution of risk factors over the area, or by population characteristics that are heterogeneously distributed over the area. We studied the distribution of leprosy cases detected by a control program to identify spatial and spatio-temporal patterns of occurrence and to search for environmental risk factors for leprosy.

**Methods:**

The houses of 11,060 leprosy cases registered in the control area during a 15-year period (1989–2003) were traced back, added to a geographic database (GIS), and plotted on digital maps. We looked for clusters of cases in space and time. Furthermore, relationships with the proximity to geographic features, such as town center, roads, rivers, and clinics, were studied.

**Results:**

Several spatio-temporal clusters were observed for voluntarily reported cases. The cases within and outside clusters did not differ in age at detection, percentage with multibacillary leprosy, or sex ratio. There was no indication of the spread from one point to other parts of the district, indicating a spatially stable endemic situation during the study period. The overall risk of leprosy in the district was not associated with roads, rivers, and leprosy clinics. The risk was highest within 1 kilometer of town centers and decreased with distance from town centers.

**Conclusion:**

The association of a risk of leprosy with the proximity to towns indicates that rural towns may play an important role in the epidemiology of leprosy in this district. Further research on the role of towns, particularly in rural areas, is warranted.

## Background

New cases of leprosy are currently found primarily in tropical regions [1,2], but the distribution within these regions is not uniform. Sixty eight percent of newly detected cases in 2005 were found in Southeast Asia, 80% of which were detected in India. In the same year, another 13% of all cases worldwide were found in Brazil. The Southeast Asian region and Brazil together accounted for 81% of all cases of leprosy detected in 2006 [2].

Within highly endemic regions, the occurrence of leprosy is also not uniformly distributed [3-5]. The distribution of leprosy in the Brazilian state of Ceará reflects socioeconomic differences within the state [4,6], whereas the explanation for the uneven distribution in another Brazilian state, São Paulo thought to be migratory movement towards the urban and developing areas in the center of the state [3]. In the Malawian Karonga district, a positive relationship between the proximity of water and leprosy incidence was previously found [5]. The relationship between open water and leprosy was hypothesized based on observed associations with rainfall and coastal populations [7,8], as well as evidence that the infectious agent, *Mycobacterium leprae*, survives longer outside the human body in humid compared to dry atmospheres [9]. In a locality with many rivers and other bodies of water, such as northwest Bangladesh, the relationship between leprosy and open water might be quite different.

Differences in the case detection rates can arise from differences in the accessibility of leprosy control facilities. In poor areas, traveling is expensive for the common people and the proximity to a leprosy control facility might increase the detection rate among the population.

A study of the spatial distribution of leprosy can contribute to the knowledge about, or identification of, the underlying risk factors for the disease and the transmission patterns of *M. leprae*. A clustering of leprosy cases at the village level was not observed in the highly endemic Nilphamari district in northwest Bangladesh [10]. In this paper we describe the spatial distribution of leprosy at the district level in the same area during the period of 1989 to 2003 and determined whether high case detection clusters were present in the district. We investigated the risk of leprosy in proximity to geographic factors that may have a relationship with the risk of leprosy, such as the environment (*i.e. *rivers and roads), a different population (*i.e. *towns), or enhanced availability of health services (*i.e. *leprosy clinics).

## Methods

### Study design

The study is a retrospective observational study on the spatial distribution of newly detected leprosy patients in northwest Bangladesh over a 15-year period.

### Study area

**Figure 1:**
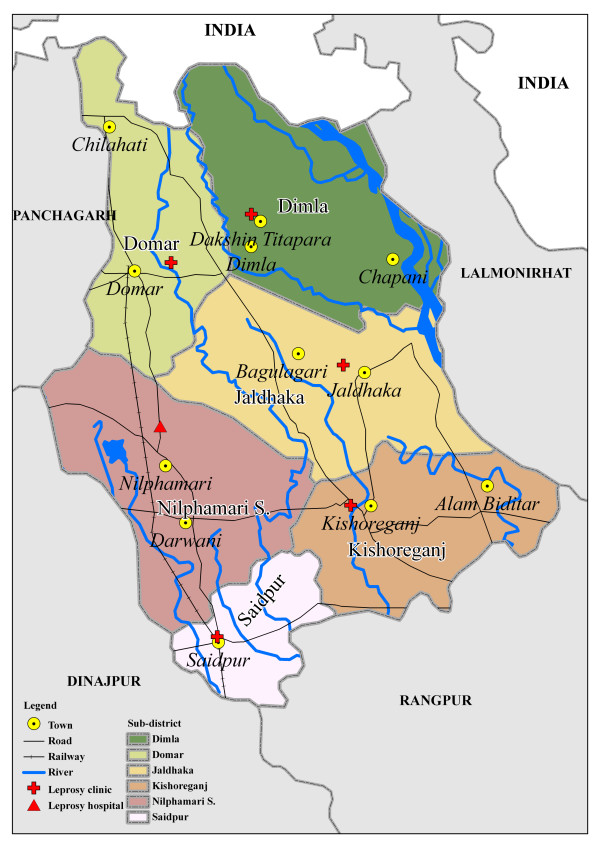
National, district, and sub-district borders, towns, clinics, rivers, roads, and railroad of Nilphamari district.

The study was conducted in the Nilphamari district at 26°00' N and 88°57' E. The district has an area of 1640.9 km^2 ^and approximately 1.5 million inhabitants [11]. The district is divided into six sub-districts. Geographical and leprosy characteristics of the sub-districts are given in Table 1. The sub-districts Nilphamari Sadar and Saidpur contain two major urban areas, also called Nilphamari and Saidpur, with Saidpur city being the largest urban area. The district is mainly rural outside these urbanized areas. The Saidpur sub-district contains a large refugee population of over 38,000 stateless Bihari refugees. The refugee camp was created near Saidpur city after the Bangladesh war for independence in the early 1970's [12]. One of the major rivers of Bangladesh, the Tista River, flows through the northeast part of the district and several smaller rivers cross the district. A map of Nilphamari district is presented in Figure 1.

**Table 1:** Leprosy, population, and geographic characteristics of the sub-districts.

Sub-district	Cases	Person-years	NCDR*	Area (km^2^)	No. Towns	No. Clinics
Nilphamari Sadar	2,501	5,003,010	0.50	249.8	2	1
Saidpur	1,654	3,375,432	0.49	339.2	1	1
Kishoregonj	1,002	4,140,829	0.24	332.8	2	1
Jaldhaka	2,215	3,791,886	0.58	338.8	2	1
Domar	1,647	2,910,790	0.57	256.3	2	1
Dimla	2,041	3,125,001	0.65	124.1	3	1

**Total**	11,060	22,346,947	0.49	1640.9	12	6

*New case detection rate per 1,000 person-years

The Danish Bangladesh Leprosy Mission (DBLM) was established in this area in 1977. Since that time, more than 95% of registered leprosy patients have been treated by DBLM. The project area also covers the neighboring districts of Rangpur, Thakurgoan, and Panchagar. The DBLM has been responsible for leprosy control in these four districts since 1994. Multidrug therapy (MDT) was completely introduced in the project area by 1991 [13].

### Study population

The study population existed of all leprosy patients diagnosed and registered between January 1, 1989 and December 31, 2003 at one of the DBLM clinics and living in Nilphamari district. Case registration was done according to the DBLM guidelines [14]; demographic data, World Health Organization (WHO) leprosy classification [15], and the mode of detection were registered. A DBLM leprosy control supervisor confirmed all cases before registration and subsequent treatment. Uncertain cases were referred to the leprosy control officer or DBLM medical officer for confirmation. An independent inspector assessed the program in 2001 and found an over-diagnosis of only 3.4% [16]. For the current study, we used the existing patient database and added spatial data.

### Mode of detection

As the data was from a running control program, the cases were detected by different modes of detection [17]: voluntary reporting, surveys, and contract tracing. Voluntarily reported cases, apart from cases presented voluntarily at a clinic, included those referred by a professional health worker or other informed respected person (*i.e. *village doctors, teachers, or health workers). Surveys consisted of school or village surveys and were performed during the entire study period. During these school or village surveys, the students of a school or the population of a certain area with an assumed high prevalence of leprosy were examined. Contact tracing was always practiced after a voluntarily reported case was confirmed and continued for 2 to 5 yearly visits, depending on the leprosy classification [14].

The occurrence of spatio-temporal clusters of high rates of detection was investigated separately for each mode of detection. The characteristics of patients within clusters were compared to patients living outside the clusters. The position of the houses of patients grouped by mode of detection was studied in relation to towns, rivers, roads, and leprosy clinics separately.

However, we focus on voluntarily reported cases because, in this control program, these cases are thought to give the best representation of the incidence of leprosy. Surveys normally tend to give a better picture of the real prevalence than voluntarily reported cases. In this control program, however, surveys were performed depending on the number of cases previously voluntarily reported in a village or school. Results of cases detected by surveys or contact tracing can be found in the supplementary information.

### Location of patients

During the current study, the houses in which patients lived at the time of diagnosis were traced back by specially trained staff. We note that this is not necessarily the location at which the patient became infected. Another possibility would have been to use location at which the patient lived when the first signs of disease were found. The location where the patient lived during diagnosis, however, could be determined more accurately, and we assume that the difference with the location at which the first signs occurred is not very different on the scale of a whole district. The coordinates were measured using a handheld GPS-unit (Geko 201 Garmin™) between January and November 2006. Cases were excluded if the patient was registered to live in a district other than Nilphamari or if the house was outside Nilphamari district according to our digital map. Finally, those whose home coordinates could not be obtained were excluded from analysis, in addition to patients for whom the mode of detection was unknown.

### Geographic and spatial data sets

A population density map with a grid cell of 30" by 30" resolution was obtained from the Gridded Population of the World version 3, beta version. [18] The population densities for each grid cell were calculated by pycnophylactic smoothing based on sub-district population counts. The population density maps were made for the population in 1995 and 2000 based upon 1991 and 2001 census data assuming an exponential growth of the population [19].

Digital maps of the administrative boundaries of the districts and sub-districts of Bangladesh were obtained from the Food and Agricultural Organization of the United Nations [20]. Road, populated places, and hydrographical data were obtained from downloadable data of the geocommunity [21].

### Statistical analyses

Case detection was plotted against time and tested for a temporal cluster [22]. A temporal cluster is a period in which case detection was higher than expected for cases randomly distributed over the study period. The likelihood that the case detection originated at random during a period was calculated assuming a Poisson distribution of cases among the population. A likelihood ratio test was used to obtain a p-value for the most likely cluster.

The area was tested for a high incidence of spatio-temporal clusters of cases separately for each detection mode using the spatio-temporal permutation test. The spatio-temporal permutation test [23] is a nonparametric test making use of the information from the case distribution. This test compares the observed number of cases during a time period in a circular area with the expected number cases if the spatial and temporal location of all cases were independent. The comparison is made for a cylindrical window with a circular geographic base and with height corresponding to the length of the time period. Both the circular base (the area) and the height of the cylinder (the time period) are flexible. The likelihood that the case detection in a certain space-time window originated by chance was calculated under the assumption that no space-time interaction exists. The expected cases in a certain area were calculated based upon the number of cases observed at that location during the entire study period and the number of cases in the whole district during that timeframe. Therefore, this method adjusts for the pure spatial and pure temporal incidence. The probability that a cluster did not originate by chance was determined by Monte Carlo hypothesis testing based upon the most likely cluster [23]. We restricted the test to clusters of a length of at least 1 year and at most 25% of the population without geographic overlap. Only space-time windows with more than the number of expected cases, *i.e. *high incidence clusters, were tested.

We compared the cases within and outside spatio-temporal clusters by calculating the distance to towns, rivers, roads, and clinics; the average age at detection; the percentage of multibacillary (MB) leprosy; and the sex ratio. For distance to towns, we took the distance measured from the center of town.

For the analysis of the proximity of towns, rivers, clinics, and roads, we used Poisson regression with a correction for over-dispersion. We calculated distances to the geographic features and used the distance and square distance as continuous variables in separate models, and we fitted a model with categorical variables of distance in categories of 1 km. We fitted a univariate model with only the explanatory variable and multivariate model with all variables (*i.e. *distance to town, river, clinic, and road).

### Software

Data entry was done in Microsoft Access 2000™ and ArcGIS^® ^9.1 was used for the visualization and processing of spatial data using a plug-in tool to count cases [24]. The temporal and spatio-temporal cluster analyses were performed with the SaTScan program, version 7.0.3 [25]. Poisson regressions were performed in R^© ^2.6.0 [26].

### Ethical clearance

The informed consent of the house inhabitants was obtained verbally. Ethical clearance was obtained from the ethical review committee of the Bangladesh Medical Research Council (reference number. BMRC/ERC/2004–2007/1397).

## Results

During the study-period, 12,602 newly detected leprosy patients were registered at clinics in Nilphamari district. We were not able to find the locations for 881 patients, and another 661 were either registered as living outside the district or found to live outside the district during this study. This left 11,060 cases for which we were able to obtain the coordinates of their house. Patients that could not be traced back, *i.e*. missing cases, were originally detected, on average, seven months earlier in the study period than the included cases. The percentage of males and year of birth were not different for missing and included cases. Forty percent of the missing cases were MB compared to twenty-eight percent of the included cases, which was a significant difference [see Additional file 1].

Of all 11,060 cases, 5170 were reported voluntarily, 1048 were found by contact tracing, and 4651 by school and village surveys. For 191 cases the detection method was unknown. The percentage of females was higher among cases detected actively than among voluntarily reported cases. The percentage of MB leprosy was higher among the voluntarily reported cases than among contact tracing, and it was lowest for cases detected during surveys.

**Figure 2:**
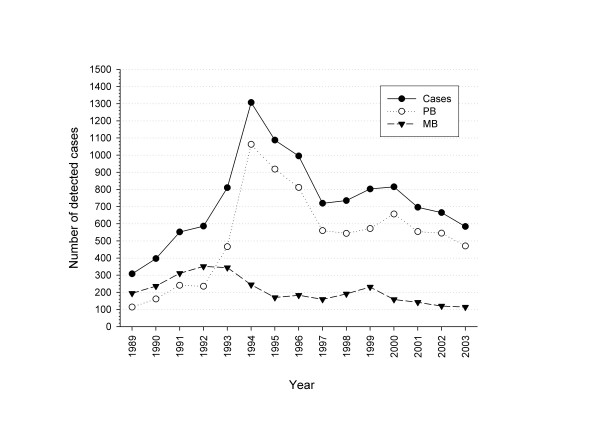
**Temporal distribution of the included cases detected in Nilphamari district between 1989 and 2003.** PB, paucibacillary; MB, multibacillary.

The detection rate increased until a peak in 1994 (Figure 2). From 1995 onwards, the number of detected cases decreased over time. The annual decline in cases was 6.44% (95% CI 4.24–8.64). A pure temporal cluster was identified between April 1994 and November 1996 consisting of primarily paucibacillary (PB) cases. This was caused by an intensification of surveys during this period (Figure 2).

**Figure 3:**
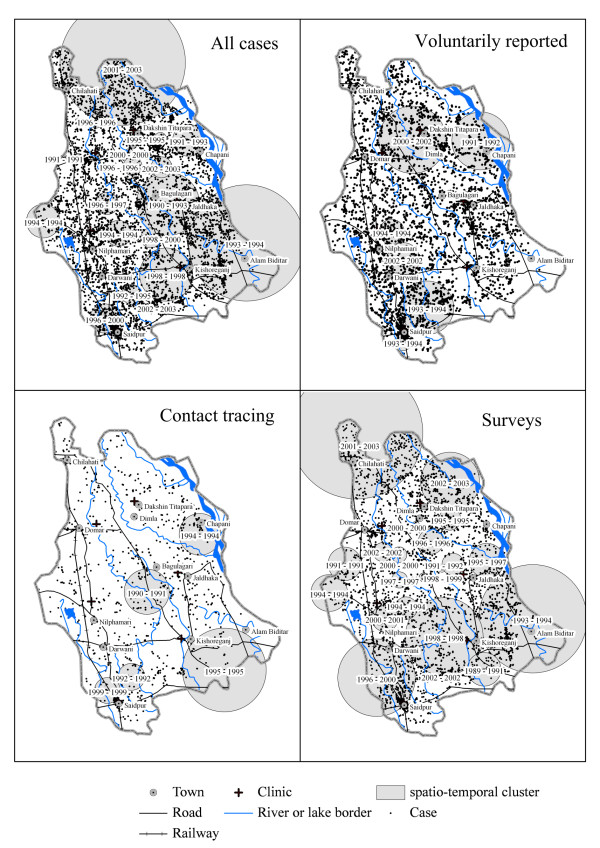
**The cases registered between 1989 and 2003 in Nilphamari district (top left).** Cases per detection mode and spatio-temporal clusters of leprosy cases detected in Nilphamari district for modes of detection, voluntarily reporting (top right), contact tracing (bottom left), and surveys (bottom right).

Many spatio-temporal clusters were found for all grouped cases and overlapped with those of the separate detection methods (Figure 3). The spatio-temporal permutation test found six clusters of voluntarily reported cases, five of contact tracing, and 20 clusters of cases found during surveys [see Table 2, Figure 3 and Additional File 2]. Most clusters had a time period of 1 or 2 years, but one cluster of survey-detected cases had a time span of 4 years. This cluster contained Saidpur city. For each detection mode, the cases within clusters did not differ in age at detection, percentage females, or the percentage of MB leprosy compared to cases outside the clusters [see Table 2 and see Additional File 2]. Furthermore, the cases within a spatio-temporal cluster did not live nearer to or further from towns, roads, clinics, or rivers for any of the detection modes. Cases within the same area were not accounted to the spatio-temporal cluster if their diagnosis was outside the timeframe of the cluster.

**Table 2:** Spatio-temporal clustering of voluntarily reported cases.

Cluster	Start	End	Cases	% females	Age at registration (yrs)	% MB
1	Jan '91	Dec '92	57	38.6% (32.4% – 44.7%)	28.4 (0 – 61.0)	70.2% (64.7% – 75.6%)
2	Jan '00	Dec '02	145	33.8% (30.2% – 37.4%)	31.2 (3.7 – 58.7)	22.8% (19.9% – 25.6%)
3	Jan '02	Dec '02	26	30.8% (22.6% – 39.0%)	37.4 (10.0 – 64.8)	26.9% (19.4% – 34.5%)
4	Jan '94	Dec '94	25	20.0% (13.7% – 26.3%)	31.9 (0 – 68.6)	40.0% (30.6% – 49.4%)
5	Jan '93	Dec '94	24	58.3% (48.6% – 68.1%)	26.8 (0 – 58.0)	20.8% (14.2% – 27.4%)
6	Jan '93	Dec '94	84	36.9% (31.9% – 41.9%)	34.1 (3.2 – 65.0)	38.1% (33.1% – 43.1%)
All clusters			361	35.7% (33.4% – 38.1%)	31.7 (1.7 – 61.6)	35.2% (32.8% – 37.5%)
Outside clusters			4809	36.0% (35.4% – 36.7%)	31.7 (0 – 65.0)	38.9% (38.2% – 39.5%)
All			5170	36.0% (35.4% – 36.6%)	31.7 (0.9 – 62.1)	38.6% (38.0% – 39.3%)

Characteristics of the most likely spatio-temporal cluster and secondary clusters that do not geographically overlap and *p *> 0.05. MB, multibacillary cases.

For voluntarily reported cases, the leprosy detection rate was higher near towns (Table 3). This seems to contradict the previous finding that cases within spatio-temporal clusters do not live nearer to towns. However, areas with a high incidence of cases throughout the entire study period do not constitute a spatio-temporal cluster. These areas can add to the risk calculated for proximity to towns. The rate decreases steeply in the first kilometers from the town. The rate of leprosy was two times lower at a distance of more than 1 to 2 kilometers from a town than the rate within 0 to 1 kilometer from town (adjusted rate ratio 0.512, 95% CI 0.387–0.677). The distance to roads was negatively related to the detection of new cases. However, the decrease in new case detection was not monotonous, with higher rates between 6 and 10 kilometers than between 2 and 6 kilometers. The rate of leprosy did not show a relationship with the distance to water [see Additional File 3]. Also, for clinics, the rate of newly detected leprosy did not change with distance [see Additional File 3].

**Table 3:** Leprosy detection rate by distance to towns.

	**Voluntarily reported**			
**Distance to town**	Univariate	95% CI	Adjusted	95% CI

Linear	0.890	(0.866 – 0.914)	0.922	(0.895 – 0.950)
Quadratic	0.990	(0.988 – 0.993)	0.993	(0.990 – 0.995)
				
Category				
0–1 km	1		1	
1–2 km	0.450	(0.342 – 0.592)	0.512	(0.387 – 0.677)
2–3 km	0.309	(0.238 – 0.403)	0.414	(0.313 – 0.549)
3–4 km	0.287	(0.221 – 0.373)	0.392	(0.294 – 0.521)
4–5 km	0.291	(0.225 – 0.376)	0.392	(0.292 – 0.525)
5–6 km	0.268	(0.206 – 0.348)	0.360	(0.264 – 0.491)
6–7 km	0.248	(0.186 – 0.329)	0.319	(0.228 – 0.446)
7–8 km	0.256	(0.190 – 0.344)	0.305	(0.215 – 0.433)
8–9 km	0.312	(0.227 – 0.429)	0.365	(0.252 – 0.527)
9–10 km	0.132	(0.072 – 0.240)	0.168	(0.090 – 0.314)
10–11 km	0.086	(0.033 – 0.222)	0.148	(0.056 – 0.392)
11–12 km	0.059	(0.012 – 0.301)	0.126	(0.025 – 0.649)
>12 km	0.082	(0.010 – 0.699)	0.270	(0.031 – 2.361)

The adjusted rate ratios are estimates from a model including distance to clinics, rivers, and roads.

## Discussion

Our first observation was a clustering of cases in a space-time window. These kind of spatio-temporal clusters depict 'outbreaks' of cases detected by voluntary reporting. Several explanations for these 'outbreaks' are possible; the most obvious is an underlying increase in the incidence of leprosy, *i.e. *a real outbreak of disease. An increased awareness among the population, however, can also cause an 'outbreak of detection'. Finally, an 'outbreak' is also observed when the population grows faster in some areas than others while the risk remains the same. Our analytical approach cannot correct for this phenomenon [23]. However, the population has grown in the whole district [11], and clusters would be expected later in the observation period, whereas the most likely cluster was found between 1991 and 1992. The detection increased dramatically in the years 1992 to 1994 due to improved organization in the leprosy control program. The most likely cluster was found prior to this period, showing that the spatio-temporal clusters both need a spatial *and *a temporal component, *i.e. *the analysis corrects for pure temporal clusters.

This leaves increased awareness and underlying increased incidence as potential explanations. If the spatio-temporal clusters are caused by an increased awareness among the population, differences would be expected in the percentage of cases with MB leprosy and the age at detection. Increased awareness results in less time between the first symptoms and reporting. A shorter delay in detection would lead to a decrease in the percentage with MB leprosy, as more PB leprosy would be found before possible self-healing or progression from PB to MB leprosy. Also, the age at detection would be lower. Neither was observed for these clusters; thus, an underlying high incidence of leprosy can be assumed responsible for this pattern. However, we found no specific determinants (*e.g. *age at detection or proportion of MB leprosy) that could explain the high incidence in the clusters.

Our second observation was with regard to the spread of disease in time. Contrary to the anecdotal observation of the introduction and subsequent spread of leprosy by Bangladeshi refugees returning from India after the war for independence in 1972, we did not observe a spread of leprosy from Saidpur city to other areas in the district, nor could we identify patterns of spread or retraction in the district during the study period. New leprosy cases appeared more or less consistently over the whole district during the 15 years of observation, indicating a stable endemic situation in space and time.

Finally, our third observation concerns geographic determinants of leprosy risk. We found a clear relation with proximity to towns, especially in the first kilometers, and the risk of leprosy. Leprosy is thought of as a rural disease [8], but our results show that rural towns, *i.e. *moderately sized towns in a rural area, contain many cases. The sharp decline within the first kilometers might indicate that it is not the distance to town, but the difference between urban and rural populations, influencing leprosy epidemiology. There are several possible explanations. First, as suggested by others, it could be the result of selective migration towards these towns [3,4]. Second, a higher awareness among the urban population is possible. Third, the circumstances in these towns are favorable for the transmission of *M. leprae*. We recommend further studies of leprosy in urban areas and towns in rural areas. If urban areas are an important source of transmission, improvements are possible by focusing more on urban leprosy control.

The rate of new leprosy cases was higher in the proximity of roads. In another setting, the risk of leprosy was found to be increased with more distance from roads. That study was based on active surveys and indicated an under-reporting with increased distance from a road [5]. In our study, all methods of detection had a higher risk of leprosy near roads; therefore, this is not the explanation for our findings. Our results can be explained by the fact that major roads connect the towns, and people living near roads also tend to live near towns. However, our maps only contained the major roads. Though roads are present in the northeastern sub-district of Domar, these are not major roads and not present in our analysis. The results for roads are not clear from our observations, and maps of all roads instead of only the major roads might give a different result.

The proximity to a clinic might increase the possibilities of voluntary reporting, but we found no relationship with the proximity to a clinic. The distance to clinics does not seem to be an obstacle for reporting leprosy.

The proximity of water has been hypothesized to be a risk factor for leprosy transmission [8], and Sterne *et al*. [5] found an association with the proximity to rivers. The increased risk would be due to the longer lifetime of *M. leprae *outside the body in a humid atmosphere, as opposed to a dry atmosphere [8,9]. In Nilphamari, a relationship with the proximity to rivers was not found. In this district, it is unlikely that the proximity to water would increase the risk of leprosy, as almost 60% of the population lives within 2 kilometers of a river, and most live much nearer to other bodies of water, such as rice paddies. Furthermore, the relative humidity does not drop below 60% and the yearly average is 80% [11].

Our study gives a thorough spatial description of the cases found during a leprosy control program, and this approach can possibly bias our results in several ways. We retrospectively traced back patients; therefore, a proportion of the cases could not be found. The demographic characteristics, including age and sex, were not different from the included patients. The missing cases, however, contained proportionally more MB cases. The reason for this is not clear, but this difference is not likely to introduce a bias in our analysis, as there is no evidence to expect that MB cases were distributed differently than PB cases. The population density maps on which we base some of the estimates were constructed by the interpolation of sub-district data [19]. The population of Nilphamari district is less smoothly distributed than suggested by these interpolated population maps. For towns, the population density will be underestimated, resulting in higher estimates for the rate of leprosy. However, these estimates are the best available population density estimates. The results obtained using this data should be interpreted cautiously, but are useful to directing new lines of research.

## Conclusion

We found that the risk of leprosy is associated with the proximity to towns, but not roads, clinics, and rivers. Although our estimates for towns may be too high due to the use of population density maps based on interpolated census data, the elevated detection of new cases for all modes of detection near and in towns indicates that rural towns play an important role in the epidemiology of leprosy in this district. Further research on the role of towns in rural areas is warranted.

## Abbreviations

GIS: geographic information system; GPS: global positioning system; MB: multibacillary; PB: paucibacillary; WHO: World Health Organization; MDT: Multi Drug Treatment; DBLM: Danish Bangladesh Leprosy Mission, Nilphamari, Bangladesh.

## Competing interests

The authors declare that they have no competing interests.

## Authors' contributions

EF was involved in all aspects of the research and drafting the manuscript. DP and SC contributed to the set-up, planning, and conduction of data collection and commented on the manuscript. JR was involved in the conception and design of the study, as well as the analysis. JR contributed considerably to the drafting of the manuscript.

## Pre-publication history

The pre-publication history for this paper can be accessed here:



## Supplementary Material

Additional file 1 Characteristics of included cases and missing data. Table providing the %-males, % MB cases, age for included cases and missing data Click here for file

Additional file 2 Spatio-temporal Clusters of cases detected by contact tracing or surveys. Results of the spatio-temporal clustering analysis, where mode of detection is contact tracing or survey. Click here for file

Additional file 3 Change of detection rate with distance to geographic features by detection method. Results of the analyses of distances to roads, rivers and clinics for voluntary reported cases, and distance to town center, roads, rivers and clinics, where mode of detection is contact tracing or survey. Click here for file
