# Supplementary material for: The spatial distribution of leprosy cases during 15 years of a leprosy control program in Bangladesh: An observational study
Source: BMC Infect Dis. 2008 Sep 23;8:126. doi: 10.1186/1471-2334-8-126 (PMC2564934; doi:10.1186/1471-2334-8-126)
Supplement: Additional file 1 — Characteristics of included cases and missing data. Table providing the %-males, % MB cases, age for included cases and missing data [file 1471-2334-8-126-S1.doc]

**Characteristics of included cases and missing data.**

**Table S1** Characteristics of included cases and missing data. The characteristics of the patients for whom no district was registered, are given in the first row. The second row gives the information of the people registered as living in Nilphamari. The included cases were only those confirmed by their spatial location. The last row gives the totals of the missing data and the included cases.

|  | Characteristic | Missing data | Included cases |  |
| --- | --- | --- | --- | --- |
| No sub-district registered | %-males  Registration date  % MB  Age at 1-1-‘04  Total | 56 %  9-July-1996  41 %  39.8  197 | 100 %  29-Dec-1995  60%  51.1  5 | * |
| Sub-district  within  Nilphamari  district | %-males  Registration date  % MB  Age at 1-1-‘04  Total | 59 %  6-Feb-1996  39 %  39.9  684 | 59 %  24-Oct-1996  28%  38.4  11055 | *  * |
| Total | %-males  Registration date  % MB  Age at 1-1-‘04  Total | 58 %  12-Mar-1996  40 %  39.9  881 | 59 %  24-Oct-1996  28%  38.4  11060 | *  * |

^*^significant difference between missing data and included cases (p <0.05)
