# Supplementary material for: The spatial distribution of leprosy cases during 15 years of a leprosy control program in Bangladesh: An observational study
Source: BMC Infect Dis. 2008 Sep 23;8:126. doi: 10.1186/1471-2334-8-126 (PMC2564934; doi:10.1186/1471-2334-8-126)
Supplement: Additional file 2 — Spatio-temporal Clusters of cases detected by contact tracing or surveys. Results of the spatio-temporal clustering analysis, where mode of detection is contact tracing or survey. [file 1471-2334-8-126-S2.doc]

**Spatio-temporal Clusters of cases detected by contact tracing or surveys**

**Table S2** Clusters of cases found by contact tracing. This table shows the characteristics of the most likely spatio-temporal cluster and secondary clusters that do have no geographical overlap and *p > 0.05*.

| **Contact tracing** | |  |  |  |  |  |  |  |  |  |  |  |  |  |  |  |  |  |  |  |  |
| --- | --- | --- | --- | --- | --- | --- | --- | --- | --- | --- | --- | --- | --- | --- | --- | --- | --- | --- | --- | --- | --- |
| Cluster | Start | End | Cases | % females |  |  |  |  |  | Age at registration | | | | |  | % MB |  |  |  |  |  |
| 1 | Jan '94 | Dec '94 | 27 | 55.6% | ( | 46.2% | - | 64.9% | ) | 31.4 | ( | 0.2 | - | 62.6 | ) | 14.8% | ( | 10.1% | - | 19.6% | ) |
| 2 | Jan '95 | Dec '95 | 18 | 66.7% | ( | 56.4% | - | 76.9% | ) | 29.4 | ( | 29.4 | - | 29.4 | ) | 5.6% | ( | 3.1% | - | 8.0% | ) |
| 3 | Jan '99 | Dec '99 | 14 | 71.4% | ( | 60.7% | - | 82.1% | ) | 17.6 | ( | 17.0 | - | 18.3 | ) | 21.4% | ( | 12.6% | - | 30.2% | ) |
| 4 | Jan '92 | Dec '92 | 11 | 54.5% | ( | 39.9% | - | 69.2% | ) | 21.5 | ( | 20.9 | - | 22.0 | ) | 54.5% | ( | 39.9% | - | 69.2% | ) |
| 5 | Jan '90 | Dec '91 | 35 | 31.4% | ( | 24.3% | - | 38.6% | ) | 23.4 | ( | 22.9 | - | 23.8 | ) | 37.1% | ( | 29.4% | - | 44.9% | ) |
| All clusters |  |  | 105 | 51.4% | ( | 46.7% | - | 56.2% | ) | 25.5 | ( | -3.2 | - | 54.2 | ) | 25.7% | ( | 22.1% | - | 29.4% | ) |
| Outside clusters | |  | 943 | 44.8% | ( | 43.2% | - | 46.3% | ) | 26.3 | ( | 25.3 | - | 27.2 | ) | 29.6% | ( | 28.3% | - | 30.9% | ) |
| All |  |  | 1048 | 45.4% | ( | 43.9% | - | 46.9% | ) | 26.2 | ( | -6.0 | - | 58.4 | ) | 29.2% | ( | 27.9% | - | 30.5% | ) |

**Table S2 – continued** Clusters of cases detected during surveys. This table shows the characteristics of the most likely spatio-temporal cluster and secondary clusters that do have no geographical overlap and *p > 0.05*.

| **Surveys** |  |  |  |  |  |  |  |  |  |  |  |  |  |  |  |  |  |  |  |  |  |
| --- | --- | --- | --- | --- | --- | --- | --- | --- | --- | --- | --- | --- | --- | --- | --- | --- | --- | --- | --- | --- | --- |
| Cluster | Start | End | Cases | % females |  |  |  |  |  | Age at registration | | | | |  | % MB |  |  |  |  |  |
| 1 | Jan '93 | Dec '94 | 255 | 43.5% | ( | 40.5% | - | 46.5% | ) | 30.8 |  | (0.0 | - | 64.6) |  | 13.3% |  | (11.9% | - | 14.8%) |  |
| 2 | Jan '96 | Dec '00 | 274 | 58.8% | ( | 55.9% | - | 61.6% | ) | 27.3 |  | (0.0 | - | 57.0) |  | 9.1% |  | (8.1% | - | 10.1%) |  |
| 3 | Jan '98 | Dec '99 | 34 | 50.0% | ( | 41.6% | - | 58.4% | ) | 34.4 |  | (3.3 | - | 65.5) |  | 11.8% |  | (8.3% | - | 15.3%) |  |
| 4 | Jan '02 | Dec '03 | 72 | 41.7% | ( | 36.1% | - | 47.3% | ) | 28.7 |  | (0.0 | - | 62.1) |  | 6.9% |  | (5.5% | - | 8.4%) |  |
| 5 | Jan '98 | Dec '98 | 58 | 50.0% | ( | 43.6% | - | 56.4% | ) | 30.1 |  | (0.0 | - | 61.7) |  | 13.8% |  | (10.7% | - | 16.9%) |  |
| 6 | Jan '89 | Dec '91 | 14 | 50.0% | ( | 36.9% | - | 63.1% | ) | 34.7 |  | (1.0 | - | 68.4) |  | 50.0% |  | (36.9% | - | 63.1%) |  |
| 7 | Jan '94 | Dec '94 | 34 | 38.2% | ( | 30.3% | - | 46.2% | ) | 32.0 |  | (0.0 | - | 76.4) |  | 14.7% |  | (10.5% | - | 18.9%) |  |
| 8 | Jan '01 | Dec '03 | 109 | 45.0% | ( | 40.3% | - | 49.6% | ) | 33.1 |  | (3.2 | - | 63.1) |  | 11.9% |  | (10.0% | - | 13.9%) |  |
| 9 | Jan '96 | Dec '96 | 21 | 47.6% | ( | 37.0% | - | 58.3% | ) | 36.8 |  | (11.5 | - | 62.1 |  | 28.6% |  | (19.8% | - | 37.3%) |  |
| 10 | Jan '02 | Dec '02 | 1 | 100.0% |  |  |  |  |  | 15.0 |  |  |  |  |  | 0.0% |  | (0.0% | - | 0.0%) |  |
| 11 | Jan '00 | Dec '00 | 11 | 54.5% | ( | 39.9% | - | 69.2% | ) | 24.0 |  | (0.0 | - | 55.6 |  | 9.1% |  | (4.2% | - | 14.0%) |  |
| 12 | Jan '95 | Dec '95 | 29 | 41.4% | ( | 32.6% | - | 50.2% | ) | 32.2 |  | (5.1 | - | 59.2 |  | 3.4% |  | (2.2% | - | 4.7%) |  |
| 13 | Jan '97 | Dec '97 | 9 | 66.7% | ( | 52.1% | - | 81.2% | ) | 19.6 |  | (0.0 | - | 55.0 |  | 0.0% |  | (0.0% | - | 0.0%) |  |
| 14 | Jan '02 | Dec '02 | 12 | 33.3% | ( | 20.8% | - | 45.9% | ) | 38.3 |  | (2.2 | - | 74.5 |  | 0.0% |  | (0.0% | - | 0.0%) |  |
| 15 | Jan '00 | Dec '01 | 27 | 70.4% | ( | 62.5% | - | 78.2% | ) | 30.9 |  | (0.0 | - | 63.1 |  | 3.7% |  | (2.4% | - | 5.0%) |  |
| 16 | Jan '94 | Dec '94 | 27 | 33.3% | ( | 25.0% | - | 41.7% | ) | 36.4 |  | (0.0 | - | 74.0 |  | 18.5% |  | (12.8% | - | 24.2%) |  |
| 17 | Jan '91 | Dec '91 | 8 | 37.5% | ( | 21.3% | - | 53.7% | ) | 27.3 |  | (0.0 | - | 61.8 |  | 37.5% |  | (21.3% | - | 53.7%) |  |
| 18 | Jan '00 | Dec '00 | 12 | 75.0% | ( | 64.4% | - | 85.6% | ) | 41.1 |  | (2.3 | - | 79.9 |  | 0.0% |  | (0.0% | - | 0.0%) |  |
| 19 | Jan '95 | Dec '97 | 41 | 43.9% | ( | 36.4% | - | 51.4% | ) | 40.4 |  | (7.3 | - | 73.5 |  | 7.3% |  | (5.2% | - | 9.4%) |  |
| 20 | Jan '91 | Dec '92 | 8 | 37.5% | ( | 21.3% | - | 53.7% | ) | 20.4 |  | (0.0 | - | 48.3 |  | 37.5% |  | (21.3% | - | 53.7%) |  |
| All clusters | |  | 1056 | 49.0% | ( | 47.5% | - | 50.5% | ) | 46.9 |  | (14.7 | - | 79.0 |  | 11.7% |  | (11.1% | - | 12.4%) |  |
| Outside clusters | |  | 3595 | 46.3% | ( | 45.4% | - | 47.1% | ) | 31.6 |  | (0.0 | - | 63.5 |  | 17.0% |  | (16.6% | - | 17.5%) |  |
| All |  |  | 4651 | 46.9% | ( | 46.2% | - | 47.6% | ) | 31.4 |  | (0.0 | - | 63.3 |  | 15.8% |  | (15.4% | - | 16.2%) |  |
