# Supplementary material for: The spatial distribution of leprosy cases during 15 years of a leprosy control program in Bangladesh: An observational study
Source: BMC Infect Dis. 2008 Sep 23;8:126. doi: 10.1186/1471-2334-8-126 (PMC2564934; doi:10.1186/1471-2334-8-126)
Supplement: Additional file 3 — Change of detection rate with distance to geographic features by detection method. Results of the analyses of distances to roads, rivers and clinics for voluntary reported cases, and distance to town center, roads, rivers and clinics, where mode of detection is contact tracing or survey. [file 1471-2334-8-126-S3.doc]

**Change of detection rate with distance to geographic features by detection method**

**Table S3** Change of voluntarily reported leprosy detection rate by distance to roads, rivers and clinics. Adjusted rate ratios are estimates from a model including distance to town ,and clinic, river or road.

|  | **Voluntarily reported** | | | | | | | |
| --- | --- | --- | --- | --- | --- | --- | --- | --- |
| **Distance to road** | Univariate | 95%-CI |  |  | Adjusted | 95%-CI |  |  |
| Linear | 0.911 | (0.894 | - | 0.929) | 0.934 | (0.915 | - | 0.953) |
| Quadratic | 0.995 | (0.993 | - | 0.996) | 0.996 | (0.999 | - | 1.002) |
|  |  |  |  |  |  |  |  |  |
| Category |  |  |  |  |  |  |  |  |
| 0-1 km | 1 |  |  |  |  |  |  |  |
| 1-2 km | 0.624 | (0.450 | - | 0.864) | 0.721 | (0.519 | - | 1.002) |
| 2-3 km | 0.348 | (0.252 | - | 0.482) | 0.489 | (0.349 | - | 0.684) |
| 3-4 km | 0.326 | (0.238 | - | 0.446) | 0.465 | (0.333 | - | 0.649) |
| 4-5 km | 0.278 | (0.203 | - | 0.381) | 0.424 | (0.301 | - | 0.597) |
| 5-6 km | 0.274 | (0.201 | - | 0.374) | 0.465 | (0.328 | - | 0.659) |
| 6-7 km | 0.308 | (0.227 | - | 0.419) | 0.555 | (0.390 | - | 0.789) |
| 7-8 km | 0.310 | (0.228 | - | 0.422) | 0.558 | (0.391 | - | 0.797) |
| 8-9 km | 0.353 | (0.258 | - | 0.481) | 0.621 | (0.436 | - | 0.884) |
| 9-10 km | 0.275 | (0.193 | - | 0.391) | 0.493 | (0.337 | - | 0.721) |
| 10-11 km | 0.150 | (0.090 | - | 0.249) | 0.275 | (0.162 | - | 0.466) |
| 11-12 km | 0.166 | (0.095 | - | 0.289) | 0.286 | (0.161 | - | 0.508) |
| 12-13 km | 0.092 | (0.044 | - | 0.194) | 0.152 | (0.072 | - | 0.325) |
| 13-14 km | 0.130 | (0.057 | - | 0.294) | 0.195 | (0.085 | - | 0.446) |
| 14-15 km | 0.124 | (0.050 | - | 0.307) | 0.197 | (0.079 | - | 0.492) |
| 15-16 km | 0.115 | (0.042 | - | 0.315) | 0.189 | (0.068 | - | 0.525) |
| 16-17 km | 0.063 | (0.014 | - | 0.289) | 0.098 | (0.021 | - | 0.457) |
| 17-18 km | 0.095 | (0.026 | - | 0.351) | 0.154 | (0.041 | - | 0.580) |
| 18-19 km | 0.092 | (0.018 | - | 0.472) | 0.156 | (0.030 | - | 0.810) |
| 19-20 km | 0.334 | (0.095 | - | 1.173) | 0.542 | (0.148 | - | 1.984) |
| >20km | 0.448 | (0.114 | - | 1.762) | 0.691 | (0.165 | - | 2.891) |

**Table S3 continued** Change of voluntarily reported leprosy detection rate by distance to roads, rivers and clinics. Adjusted rate ratios are estimates from a model including distance to town ,and clinic, river or road.

|  | **Voluntarily reported** | | | | | | | |
| --- | --- | --- | --- | --- | --- | --- | --- | --- |
| **Distance to river** | Univariate | 95%-CI |  |  | Adjusted | 95%-CI |  |  |
| Linear | 1.028 | (0.988 | - | 1.070) | 1.033 | (0.992 | - | 1.075) |
| Quadratic | 0.998 | (0.990 | - | 1.005) | 0.998 | (0.996 | - | 1.012) |
|  |  |  |  |  |  |  |  |  |
| Category |  |  |  |  |  |  |  |  |
| 0-1 km | 1 |  |  |  |  |  |  |  |
| 1-2 km | 1.417 | (1.220 | - | 1.645) | 1.295 | (1.112 | - | 1.509) |
| 2-3 km | 1.342 | (1.124 | - | 1.603) | 1.278 | (1.066 | - | 1.533) |
| 3-4 km | 1.403 | (1.143 | - | 1.721) | 1.280 | (1.037 | - | 1.580) |
| 4-5 km | 1.142 | (0.869 | - | 1.501) | 1.083 | (0.821 | - | 1.429) |
| 5-6 km | 0.790 | (0.508 | - | 1.227) | 0.868 | (0.556 | - | 1.356) |
| 6-7 km | 0.914 | (0.394 | - | 2.121) | 0.870 | (0.371 | - | 2.039) |
| 7-8 km | 0.373 | (0.044 | - | 3.147) | 0.348 | (0.041 | - | 2.958) |
|  |  |  |  |  |  |  |  |  |

**Table S3 continued** Change of Voluntarily reported leprosy detection rate by distance to roads, rivers and clinics. Adjusted rate ratios are estimates from a model including distance to town ,and clinic, river or road.

| **Distance to clinic** | Univariate | 95%-CI |  |  | Adjusted | 95%-CI |  |  |
| --- | --- | --- | --- | --- | --- | --- | --- | --- |
| Linear | 0.963 | (0.939 | - | 0.987) | 1.006 | (0.981 | - | 1.033) |
| Quadratic | 0.997 | (0.995 | - | 0.999) | 1.000 | (0.999 | - | 1.004) |
|  |  |  |  |  |  |  |  |  |
| Category |  |  |  |  |  |  |  |  |
| 0-1 km | 1 |  |  |  |  |  |  |  |
| 1-2 km | 0.634 | (0.537 | - | 0.749) | 0.787 | (0.662 | - | 0.937) |
| 2-3 km | 0.763 | (0.640 | - | 0.910) | 0.978 | (0.811 | - | 1.179) |
| 3-4 km | 0.681 | (0.545 | - | 0.850) | 0.857 | (0.680 | - | 1.081) |
| 4-5 km | 0.821 | (0.636 | - | 1.060) | 0.992 | (0.761 | - | 1.293) |
| 5-6 km | 0.858 | (0.601 | - | 1.225) | 0.996 | (0.690 | - | 1.438) |
| 6-7 km | 1.034 | (0.698 | - | 1.531) | 1.362 | (0.904 | - | 2.053) |
| 7-8 km | 0.937 | (0.598 | - | 1.470) | 1.202 | (0.762 | - | 1.896) |
| 8-9 km | 0.576 | (0.311 | - | 1.066) | 0.826 | (0.443 | - | 1.538) |
| 9-10 km | 0.671 | (0.367 | - | 1.228) | 1.066 | (0.577 | - | 1.969) |
| 10-11km | 0.552 | (0.261 | - | 1.166) | 0.777 | (0.365 | - | 1.653) |
| 11-12 km | 1.114 | (0.602 | - | 2.062) | 1.696 | (0.904 | - | 3.180) |
| 12-13 km | 0.767 | (0.359 | - | 1.638) | 1.184 | (0.545 | - | 2.569) |
| 13-14 km | 0.182 | (0.027 | - | 1.227) | 0.441 | (0.064 | - | 3.014) |
| 14-15 km | 0.048 | (0.001 | - | 3.371) | 0.192 | (0.003 | - | 13.854) |
| >15 km | 0.142 | (0.007 | - | 2.895) | 0.553 | (0.026 | - | 11.683) |

**Table S4** Change of survey leprosy detection rate by distance to roads, rivers and clinics. Adjusted rate ratios are estimates from a model including distance to town ,and clinic, river or road.

| **Distance to town** | Univariate | 95%-CI | | | | | Adjusted | | 95%-CI | | | | |  |
| --- | --- | --- | --- | --- | --- | --- | --- | --- | --- | --- | --- | --- | --- | --- |
| Linear | 0.942 | ( | 0.915 | - | 0.970 | ) | | 0.945 | | ( | 0.915 | - | 0.976) | |
| Quadratic | 0.996 | ( | 0.993 | - | 0.998 | ) | | 0.996 | | ( | 0.993 | - | 0.999) | |
| Category |  |  |  |  |  |  | |  | |  |  |  |  | |
| 0-1 km | 1 |  |  |  |  |  | |  | |  |  |  |  | |
| 1-2 km | 0.556 | ( | 0.390 | - | 0.792 | ) | | 0.616 | | ( | 0.430 | - | 0.882) | |
| 2-3 km | 0.411 | ( | 0.294 | - | 0.577 | ) | | 0.525 | | ( | 0.368 | - | 0.749) | |
| 3-4 km | 0.422 | ( | 0.303 | - | 0.588 | ) | | 0.541 | | ( | 0.379 | - | 0.774) | |
| 4-5 km | 0.413 | ( | 0.297 | - | 0.573 | ) | | 0.505 | | ( | 0.350 | - | 0.728) | |
| 5-6 km | 0.374 | ( | 0.268 | - | 0.523 | ) | | 0.432 | | ( | 0.294 | - | 0.634) | |
| 6-7 km | 0.388 | ( | 0.274 | - | 0.551 | ) | | 0.423 | | ( | 0.282 | - | 0.632) | |
| 7-8 km | 0.408 | ( | 0.285 | - | 0.585 | ) | | 0.407 | | ( | 0.268 | - | 0.616) | |
| 8-9 km | 0.460 | ( | 0.313 | - | 0.677 | ) | | 0.412 | | ( | 0.265 | - | 0.639) | |
| 9-10 km | 0.297 | ( | 0.167 | - | 0.529 | ) | | 0.285 | | ( | 0.155 | - | 0.524) | |
| 10-11 km | 0.172 | ( | 0.068 | - | 0.434 | ) | | 0.216 | | ( | 0.084 | - | 0.558) | |
| 11-12 km | 0.319 | ( | 0.120 | - | 0.845 | ) | | 0.492 | | ( | 0.180 | - | 1.342) | |
| >12 km | 0.354 | ( | 0.087 | - | 1.446 | ) | | 0.803 | | ( | 0.186 | - | 3.468) | |
|  |  |  |  |  |  |  | |  | |  |  |  |  | |

**Table S4 continued**

| **Distance to clinic** | Univariate | 95%-CI | | | | | Adjusted | | 95%-CI | | | | |  |
| --- | --- | --- | --- | --- | --- | --- | --- | --- | --- | --- | --- | --- | --- | --- |
| Linear | 1.032 | ( | 1.009 | - | 1.056 | ) | | 1.065 | | ( | 1.039 | - | 1.091) | |
| Quadratic | 1.002 | ( | 1.000 | - | 1.004 | ) | | 1.004 | | ( | 0.999 | - | 1.005) | |
| Category |  |  |  |  |  |  | |  | |  |  |  |  | |
| 0-1 km | 1 |  |  | - |  |  | |  | |  |  |  |  | |
| 1-2 km | 0.631 | ( | 0.517 | - | 0.770 | ) | | 0.732 | | ( | 0.595 | - | 0.900) | |
| 2-3 km | 0.833 | ( | 0.679 | - | 1.022 | ) | | 1.000 | | ( | 0.806 | - | 1.240) | |
| 3-4 km | 0.780 | ( | 0.606 | - | 1.004 | ) | | 0.921 | | ( | 0.709 | - | 1.197) | |
| 4-5 km | 0.916 | ( | 0.684 | - | 1.226 | ) | | 1.034 | | ( | 0.764 | - | 1.399) | |
| 5-6 km | 1.187 | ( | 0.822 | - | 1.716 | ) | | 1.293 | | ( | 0.887 | - | 1.887) | |
| 6-7 km | 1.660 | ( | 1.137 | - | 2.423 | ) | | 2.211 | | ( | 1.501 | - | 3.255) | |
| 7-8 km | 1.653 | ( | 1.094 | - | 2.498 | ) | | 1.923 | | ( | 1.263 | - | 2.929) | |
| 8-9 km | 1.757 | ( | 1.139 | - | 2.709 | ) | | 2.094 | | ( | 1.343 | - | 3.265) | |
| 9-10 km | 1.312 | ( | 0.777 | - | 2.216 | ) | | 1.756 | | ( | 1.024 | - | 3.011) | |
| 10-11km | 1.251 | ( | 0.685 | - | 2.282 | ) | | 1.515 | | ( | 0.821 | - | 2.796) | |
| 11-12 km | 1.898 | ( | 1.073 | - | 3.357 | ) | | 2.324 | | ( | 1.294 | - | 4.176) | |
| 12-13 km | 1.075 | ( | 0.497 | - | 2.324 | ) | | 1.425 | | ( | 0.648 | - | 3.136) | |
| 13-14 km | 0.318 | ( | 0.056 | - | 1.798 | ) | | 0.678 | | ( | 0.118 | - | 3.910) | |
| 14-15 km | 0.059 | ( | 0.001 | - | 5.760 | ) | | 0.189 | | ( | 0.002 | - | 18.657) | |
| >15 km | 0.089 | ( | 0.001 | - | 8.605 | ) | | 0.284 | | ( | 0.003 | - | 28.217) | |
|  |  |  |  |  |  |  | |  | |  |  |  |  | |

**Table S4 continued**

| **Distance to road** | Univariate | 95%-CI | | | | | | Adjusted | | 95%-CI | | | |
| --- | --- | --- | --- | --- | --- | --- | --- | --- | --- | --- | --- | --- | --- |
| Linear | 0.954 | ( | 0.934 | - | 0.973 | ) | 0.955 | | ( | | 0.934 | - | 0.976) |
| Quadratic | 0.997 | ( | 0.995 | - | 0.998 | ) | 0.997 | | ( | | 0.999 | - | 1.002) |
| Category |  |  |  |  |  |  |  | |  | |  |  |  |
| 0-1 km | 1 |  |  | - |  |  |  | |  | |  |  |  |
| 1-2 km | 0.838 | ( | 0.545 | - | 1.288 | ) | 0.923 | | ( | | 0.598 | - | 1.423) |
| 2-3 km | 0.434 | ( | 0.281 | - | 0.670 | ) | 0.560 | | ( | | 0.359 | - | 0.875) |
| 3-4 km | 0.375 | ( | 0.245 | - | 0.575 | ) | 0.463 | | ( | | 0.296 | - | 0.725) |
| 4-5 km | 0.405 | ( | 0.268 | - | 0.613 | ) | 0.530 | | ( | | 0.340 | - | 0.825) |
| 5-6 km | 0.401 | ( | 0.266 | - | 0.603 | ) | 0.572 | | ( | | 0.365 | - | 0.895) |
| 6-7 km | 0.503 | ( | 0.337 | - | 0.749 | ) | 0.751 | | ( | | 0.481 | - | 1.174) |
| 7-8 km | 0.546 | ( | 0.367 | - | 0.812 | ) | 0.802 | | ( | | 0.513 | - | 1.255) |
| 8-9 km | 0.664 | ( | 0.446 | - | 0.988 | ) | 0.961 | | ( | | 0.618 | - | 1.495) |
| 9-10 km | 0.466 | ( | 0.301 | - | 0.723 | ) | 0.664 | | ( | | 0.415 | - | 1.061) |
| 10-11 km | 0.284 | ( | 0.161 | - | 0.502 | ) | 0.370 | | ( | | 0.204 | - | 0.670) |
| 11-12 km | 0.286 | ( | 0.152 | - | 0.538 | ) | 0.366 | | ( | | 0.190 | - | 0.704) |
| 12-13 km | 0.192 | ( | 0.090 | - | 0.412 | ) | 0.226 | | ( | | 0.103 | - | 0.497) |
| 13-14 km | 0.276 | ( | 0.121 | - | 0.627 | ) | 0.297 | | ( | | 0.128 | - | 0.687) |
| 14-15 km | 0.295 | ( | 0.125 | - | 0.698 | ) | 0.391 | | ( | | 0.163 | - | 0.936) |
| 15-16 km | 0.192 | ( | 0.064 | - | 0.582 | ) | 0.284 | | ( | | 0.093 | - | 0.870) |
| 16-17 km | 0.105 | ( | 0.020 | - | 0.552 | ) | 0.147 | | ( | | 0.028 | - | 0.781) |
| 17-18 km | 0.159 | ( | 0.038 | - | 0.661 | ) | 0.227 | | ( | | 0.054 | - | 0.961) |
| 18-19 km | 0.111 | ( | 0.014 | - | 0.884 | ) | 0.152 | | ( | | 0.019 | - | 1.234) |
| 19-20 km | 0.327 | ( | 0.056 | - | 1.912 | ) | 0.340 | | ( | | 0.056 | - | 2.055) |
| >20km | 0.075 | ( | 0.001 | - | 7.382 | ) | 0.066 | | ( | | 0.001 | - | 6.587) |

**Table S4 continued**

| **Distance to river** | Univariate | 95%-CI | | | | | Adjusted | | 95%-CI | | | |
| --- | --- | --- | --- | --- | --- | --- | --- | --- | --- | --- | --- | --- |
| Linear | 1.042 | ( | 0.997 | - | 1.089 | ) | | 1.051 | ( | 1.005 | - | 1.100) |
| Quadratic | 1.000 | ( | 0.991 | - | 1.008 | ) | | 1.001 | ( | 0.996 | - | 1.013) |
| Category |  |  |  |  |  |  | |  |  |  |  |  |
| 0-1 km | 1 |  |  | - |  |  | |  |  |  |  |  |
| 1-2 km | 1.340 | ( | 1.129 | - | 1.590 | ) | | 1.293 | ( | 1.085 | - | 1.540) |
| 2-3 km | 1.325 | ( | 1.083 | - | 1.620 | ) | | 1.264 | ( | 1.029 | - | 1.554) |
| 3-4 km | 1.656 | ( | 1.332 | - | 2.059 | ) | | 1.465 | ( | 1.170 | - | 1.835) |
| 4-5 km | 0.969 | ( | 0.696 | - | 1.349 | ) | | 0.902 | ( | 0.644 | - | 1.262) |
| 5-6 km | 0.944 | ( | 0.597 | - | 1.493 | ) | | 1.078 | ( | 0.677 | - | 1.715) |
| 6-7 km | 0.661 | ( | 0.217 | - | 2.018 | ) | | 0.568 | ( | 0.185 | - | 1.749) |
| 7-8 km | 0.516 | ( | 0.067 | - | 4.006 | ) | | 0.437 | ( | 0.056 | - | 3.427) |
|  |  |  |  |  |  |  | |  |  |  |  |  |

**Table S5** Change of contact tracing leprosy detection rate by distance to roads, rivers and clinics. Adjusted rate ratios are estimates from a model including distance to town ,and clinic, river or road.

| **Distance to town** | Univariate | 95%-CI | | | |  | | Adjusted | 95%-CI | | | | | |
| --- | --- | --- | --- | --- | --- | --- | --- | --- | --- | --- | --- | --- | --- | --- |
| Linear | 0.900 | ( | 0.860 | - | 0.942 | ) | 0.892 | | ( | 0.849 | - | 0.937 | ) |  |
| Quadratic | 0.992 | ( | 0.988 | - | 0.996 | ) | 0.992 | | ( | 0.998 | - | 1.007 | ) |  |
| Category |  |  |  |  |  |  |  | |  |  |  |  |  |  |
| 0-1 km | 1 |  |  |  |  |  |  | |  |  |  |  |  |  |
| 1-2 km | 0.451 | ( | 0.285 | - | 0.715 | ) | 0.503 | | ( | 0.314 | - | 0.806 | ) |  |
| 2-3 km | 0.292 | ( | 0.187 | - | 0.457 | ) | 0.355 | | ( | 0.220 | - | 0.572 | ) |  |
| 3-4 km | 0.257 | ( | 0.164 | - | 0.402 | ) | 0.279 | | ( | 0.170 | - | 0.458 | ) |  |
| 4-5 km | 0.287 | ( | 0.186 | - | 0.443 | ) | 0.302 | | ( | 0.184 | - | 0.497 | ) |  |
| 5-6 km | 0.207 | ( | 0.130 | - | 0.330 | ) | 0.201 | | ( | 0.117 | - | 0.346 | ) |  |
| 6-7 km | 0.297 | ( | 0.187 | - | 0.470 | ) | 0.265 | | ( | 0.153 | - | 0.459 | ) |  |
| 7-8 km | 0.305 | ( | 0.189 | - | 0.491 | ) | 0.237 | | ( | 0.133 | - | 0.420 | ) |  |
| 8-9 km | 0.267 | ( | 0.153 | - | 0.467 | ) | 0.185 | | ( | 0.098 | - | 0.352 | ) |  |
| 9-10 km | 0.144 | ( | 0.055 | - | 0.381 | ) | 0.110 | | ( | 0.040 | - | 0.303 | ) |  |
| 10-11 km | 0.078 | ( | 0.015 | - | 0.415 | ) | 0.077 | | ( | 0.014 | - | 0.415 | ) |  |
| 11-12 km | 0.081 | ( | 0.008 | - | 0.838 | ) | 0.100 | | ( | 0.009 | - | 1.046 | ) |  |
| >12 km | 0.296 | ( | 0.044 | - | 2.008 | ) | 0.580 | | ( | 0.080 | - | 4.209 | ) |  |

**Table S5 continued**

| **Distance to clinic** | Univariate | 95%-CI | | | |  | | Adjusted | 95%-CI | | | | |
| --- | --- | --- | --- | --- | --- | --- | --- | --- | --- | --- | --- | --- | --- |
| Linear | 1 |  |  | - |  |  |  | |  |  |  |  |  |
| Quadratic | 0.602 | ( | 0.446 | - | 0.813 | ) | 0.738 | | ( | 0.539 | - | 1.011 | ) |
| Category | 0.787 | ( | 0.578 | - | 1.073 | ) | 0.995 | | ( | 0.716 | - | 1.381 | ) |
| 0-1 km | 0.716 | ( | 0.487 | - | 1.054 | ) | 0.918 | | ( | 0.614 | - | 1.373 | ) |
| 1-2 km | 1.009 | ( | 0.666 | - | 1.528 | ) | 1.249 | | ( | 0.809 | - | 1.927 | ) |
| 2-3 km | 1.022 | ( | 0.570 | - | 1.833 | ) | 1.193 | | ( | 0.655 | - | 2.172 | ) |
| 3-4 km | 1.785 | ( | 1.037 | - | 3.071 | ) | 2.363 | | ( | 1.337 | - | 4.178 | ) |
| 4-5 km | 1.506 | ( | 0.795 | - | 2.853 | ) | 1.712 | | ( | 0.893 | - | 3.284 | ) |
| 5-6 km | 1.584 | ( | 0.808 | - | 3.107 | ) | 2.044 | | ( | 1.025 | - | 4.077 | ) |
| 6-7 km | 0.994 | ( | 0.409 | - | 2.414 | ) | 1.471 | | ( | 0.592 | - | 3.656 | ) |
| 7-8 km | 1.624 | ( | 0.740 | - | 3.567 | ) | 2.348 | | ( | 1.047 | - | 5.266 | ) |
| 8-9 km | 2.207 | ( | 1.005 | - | 4.847 | ) | 2.999 | | ( | 1.329 | - | 6.767 | ) |
| > 10 km | 1.163 | ( | 0.387 | - | 3.493 | ) | 1.608 | | ( | 0.520 | - | 4.967 | ) |

**Table S5 continued**

| **Distance to road** | Univariate | 95%-CI | | | |  | Adjusted | 95%-CI | | | | |
| --- | --- | --- | --- | --- | --- | --- | --- | --- | --- | --- | --- | --- |
| Linear | 0.962 | ( | 0.933 | - | 0.991 | ) | 0.975 | ( | 0.944 | - | 1.006 | ) |
| Quadratic | 0.997 | ( | 0.996 | - | 0.999 | ) | 0.998 | ( | 0.999 | - | 1.003 | ) |
| Category |  |  |  |  |  |  |  |  |  |  |  |  |
| 0-1 km | 1 |  |  | - |  |  |  |  |  |  |  |  |
| 1-2 km | 0.645 | ( | 0.347 | - | 1.199 | ) | 0.768 | ( | 0.411 | - | 1.435 | ) |
| 2-3 km | 0.310 | ( | 0.164 | - | 0.586 | ) | 0.484 | ( | 0.251 | - | 0.933 | ) |
| 3-4 km | 0.365 | ( | 0.202 | - | 0.657 | ) | 0.612 | ( | 0.327 | - | 1.146 | ) |
| 4-5 km | 0.285 | ( | 0.156 | - | 0.521 | ) | 0.528 | ( | 0.275 | - | 1.011 | ) |
| 5-6 km | 0.341 | ( | 0.192 | - | 0.607 | ) | 0.729 | ( | 0.382 | - | 1.388 | ) |
| 6-7 km | 0.448 | ( | 0.256 | - | 0.782 | ) | 0.984 | ( | 0.520 | - | 1.864 | ) |
| 7-8 km | 0.403 | ( | 0.228 | - | 0.711 | ) | 0.872 | ( | 0.455 | - | 1.673 | ) |
| 8-9 km | 0.555 | ( | 0.318 | - | 0.971 | ) | 1.219 | ( | 0.650 | - | 2.285 | ) |
| 9-10 km | 0.486 | ( | 0.267 | - | 0.885 | ) | 1.034 | ( | 0.543 | - | 1.971 | ) |
| 10-11 km | 0.303 | ( | 0.141 | - | 0.653 | ) | 0.547 | ( | 0.244 | - | 1.223 | ) |
| 11-12 km | 0.197 | ( | 0.073 | - | 0.533 | ) | 0.327 | ( | 0.117 | - | 0.911 | ) |
| 12-13 km | 0.203 | ( | 0.073 | - | 0.566 | ) | 0.316 | ( | 0.110 | - | 0.910 | ) |
| 13-14 km | 0.189 | ( | 0.050 | - | 0.712 | ) | 0.255 | ( | 0.067 | - | 0.974 | ) |
| 14-15 km | 0.130 | ( | 0.024 | - | 0.710 | ) | 0.204 | ( | 0.037 | - | 1.136 | ) |
| 15-16 km | 0.302 | ( | 0.087 | - | 1.056 | ) | 0.593 | ( | 0.165 | - | 2.130 | ) |
| > 16 km | 0.197 | ( | 0.036 | - | 1.078 | ) | 0.406 | ( | 0.072 | - | 2.291 | ) |

**Table S5 continued**

| **Distance to river** | Univariate | 95%-CI | | | |  | | Adjusted | | 95%-CI | | | | | |  |
| --- | --- | --- | --- | --- | --- | --- | --- | --- | --- | --- | --- | --- | --- | --- | --- | --- |
| Linear | 0.981 | ( | 0.915 | - | 1.052 | | ) | | 0.991 | | ( | 0.923 | - | 1.064 | ) | |
| Quadratic | 0.989 | ( | 0.976 | - | 1.003 | | ) | | 0.991 | | ( | 0.993 | - | 1.022 | ) | |
| Category |  |  |  |  |  | |  | |  | |  |  |  |  |  | |
| 0-1 km | 1 |  |  | - |  | |  | |  | |  |  |  |  |  | |
| 1-2 km | 1.359 | ( | 1.062 | - | 1.740 | | ) | | 1.277 | | ( | 0.992 | - | 1.644 | ) | |
| 2-3 km | 1.117 | ( | 0.821 | - | 1.520 | | ) | | 1.019 | | ( | 0.743 | - | 1.399 | ) | |
| 3-4 km | 1.378 | ( | 0.984 | - | 1.930 | | ) | | 1.189 | | ( | 0.839 | - | 1.686 | ) | |
| 4-5 km | 0.546 | ( | 0.294 | - | 1.015 | | ) | | 0.509 | | ( | 0.272 | - | 0.952 | ) | |
| 5-6 km | 0.949 | ( | 0.489 | - | 1.840 | | ) | | 1.150 | | ( | 0.587 | - | 2.253 | ) | |
| 6-7 km | 0.482 | ( | 0.073 | - | 3.192 | | ) | | 0.418 | | ( | 0.062 | - | 2.795 | ) | |
|  |  |  |  |  |  | |  | |  | |  |  |  |  |  | |
